# Retained Surgicel mimicking renal tumor recurrence: A diagnostic pitfall following partial nephrectomy – A case report

**DOI:** 10.1016/j.ijscr.2025.111774

**Published:** 2025-08-12

**Authors:** Anahita Ansari Djafari, Sina Samenezhad, Seyyed Ali Hojjati, Azadeh Rakhsha, Dorna Rafighi

**Affiliations:** a Urology Department, School of Medicine, Shahid Beheshti University of Medical Sciences, Tehran, Iran; b Laser application in medical sciences research center, Shahid Beheshti university of medical Sciences, Tehran, Iran; c Department of Pathology, Shohada-e-tajrish Educational Hospital, School of Medicine, Shahid Beheshti University of Medical Sciences, Tehran, Iran; d Department of Microbiology, Faculty of Basic Sciences, Research Sciences Branch, Islamic Azad University, Tehran, Iran

**Keywords:** Partial nephrectomy, Surgicel, Oxidized cellulose, Renal tumor recurrence, Foreign body granuloma, Imaging pitfall

## Abstract

**Introduction and importance:**

Partial nephrectomy is the standard treatment for localized renal cell carcinoma, often using oxidized cellulose (Surgicel®) for hemostasis. Retained Surgicel® may mimic tumor recurrence on imaging, posing a critical diagnostic challenge with potential for unnecessary interventions.

**Case presentation:**

We report a 54-year-old female with chromophobe renal cell carcinoma who underwent open partial nephrectomy with Surgicel® placement. Two years later, imaging revealed a suspicious renal mass suggesting tumor recurrence. Surgical excision and histopathology identified a foreign body granulomatous reaction to retained Surgicel®, with no malignancy detected.

**Clinical discussion:**

Surgicel® typically resorbs within weeks but can occasionally cause granulomatous reactions appearing as enhancing masses with diffusion restriction on MRI, mimicking malignancy. Awareness of this entity and correlation with surgical history are essential. T2-weighted MRI assists differentiation, and biopsy confirms diagnosis, preventing unnecessary surgery.

**Conclusion:**

Retained Surgicel® is a rare but important cause of false-positive tumor recurrence after partial nephrectomy. Accurate diagnosis through imaging, clinical correlation, and multidisciplinary communication can avoid misdiagnosis and unwarranted invasive procedures.

## Highlights

• Retained Surgicel® can mimic renal tumor recurrence on imaging after partial nephrectomy.
• Surgicel® granulomas show contrast enhancement and diffusion restriction, causing diagnostic confusion.
• T2-weighted MRI helps differentiate retained Surgicel® from true tumor recurrence or hematoma.
• Awareness prevents unnecessary anxiety, invasive tests, and unwarranted surgeries.
• Thorough surgical history and interdisciplinary communication are key to accurate diagnosis

## 1 Introduction

Partial nephrectomy is the standard for localized Renal Cell Carcinoma when feasible, offering renal function preservation at the cost of increased surgical complexity. While minimally invasive PN has gained popularity, open partial nephrectomy remains valuable, particularly in complex cases such as solitary kidneys, multifocal tumors, or prior surgeries [1]. Use of oxidized cellulose hemostats provided effective hemostasis and facilitated closure of large renal parenchymal defects after tumor resection, without evidence of local ischemia or parenchymal necrosis [2]. Patients with retained surgical items may present with a wide range of symptoms, including abdominal pain, abscess formation, nausea, vomiting, wound complications, or palpable masses, although many remain asymptomatic. The clinical presentation often correlates with the anatomical location of the retained item, underscoring the importance of compartment-specific evaluation. Notably, retained surgical items should be considered in the differential diagnosis of unexplained masses or delayed postoperative complications—even years or decades after the initial procedure [3]. This case report highlights a rare but critical diagnostic pitfall where retained Surgicel, a commonly used hemostatic agent, mimicked local tumor recurrence on imaging studies, leading to significant diagnostic confusion. Such mimicry can result in unnecessary anxiety, invasive investigations, or even unwarranted interventions if not recognized promptly. By sharing this case, we aim to raise awareness among clinicians and radiologists about the potential for oxidized cellulose to present as a pseudo-tumor, emphasizing the need for careful correlation of clinical history, surgical details, and imaging findings to avoid misdiagnosis and optimize patient management.

## 2 Case presentation

A 54-year-old female with a history of chromophobe renal cell carcinoma of the left kidney underwent open partial nephrectomy two years prior to presentation. The tumor measured 34 × 31 mm and was located in the inferior pole of the kidney. Histopathological examination confirmed chromophobe renal cell carcinoma staged as pT1a, with negative surgical margins. An additional tumor bed resection was also performed, which showed no residual tumor. During the procedure, oxidized regenerated cellulose (Surgicel®) was placed at the base of the resection site to aid hemostasis.

Postoperative recovery was uneventful. The patient remained asymptomatic with no abdominal pain, nausea, vomiting, or urinary complaints. A follow-up abdominopelvic ultrasound performed six months after surgery revealed no abnormalities. At 12 months, the patient developed a bulge at the surgical site, clinically diagnosed as an incisional hernia. A non-contrast abdominopelvic computed tomography (CT) scan was performed, revealing a 37 × 39 mm soft tissue density adjacent to the inferomedial aspect of the left kidney, extending into the surgical defect in the lateral abdominal wall. Additionally, a large left lateral abdominal hernia measuring 70 × 106 mm was noted. The remaining abdominal and pelvic organs were unremarkable.

**Fig. 1.**
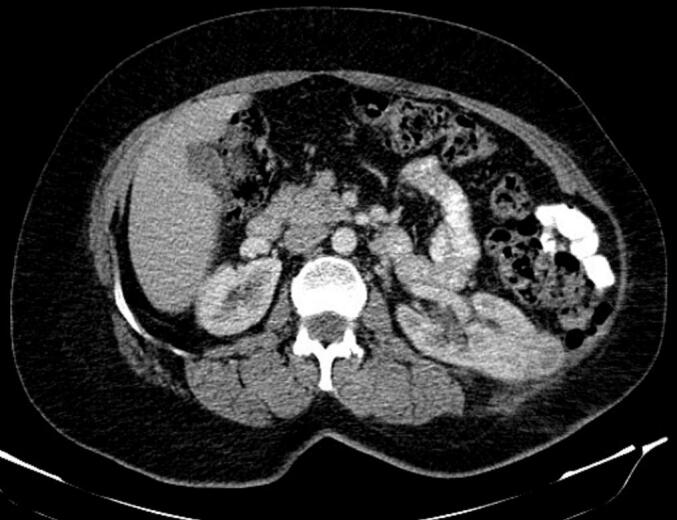

**Fig. 2.**
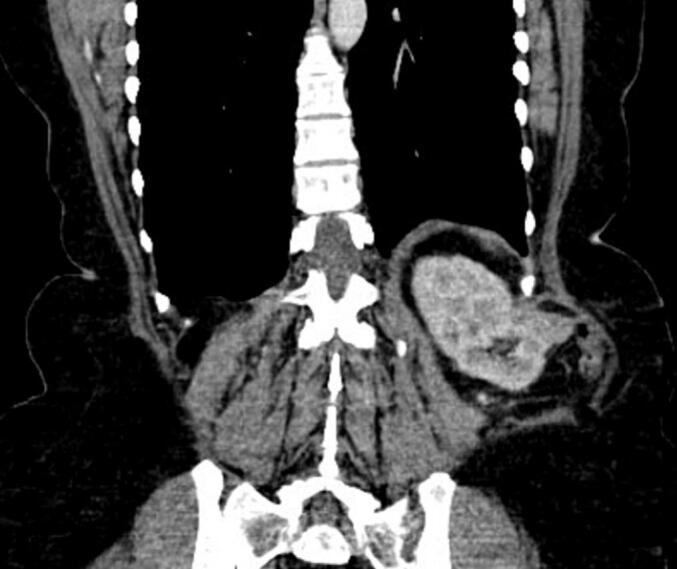

To further characterize the renal lesion, a contrast-enhanced CT scan was obtained. It demonstrated a cortical, heterogeneously enhancing mass measuring 45 × 38 × 37 mm at the upper pole of the left kidney, with adherence to the posterior renal fascia. There was no evidence of renal vein thrombosis. The previously described lateral lumbar hernia containing bowel loops was again noted. (Fig. 1., Fig. 2.) In light of the concern for local tumor recurrence, an abdominal MRI with and without gadolinium contrast was performed.

**Fig. 3.**
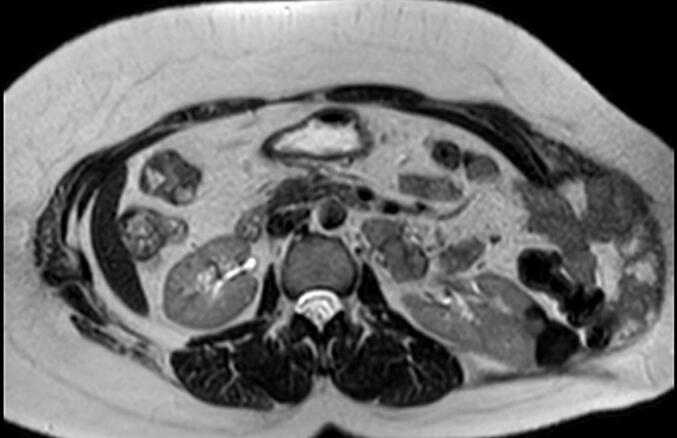

**Fig. 4.**
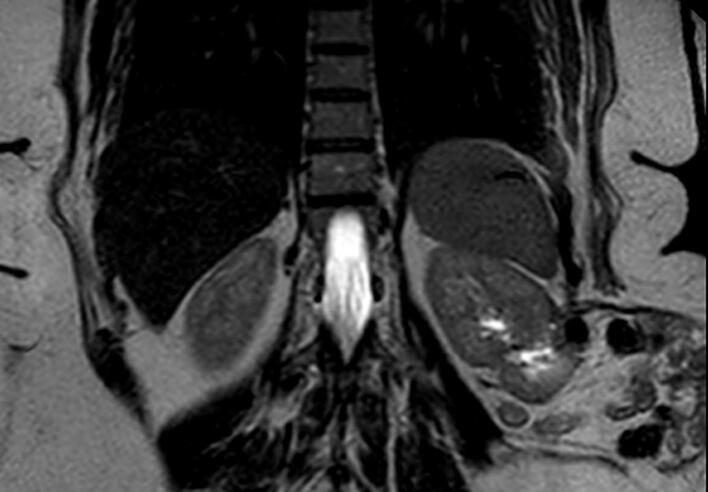

**Fig. 5.**
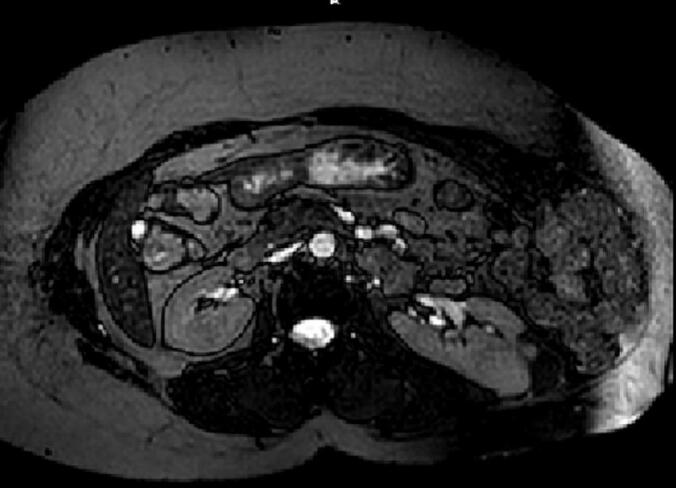

**Fig. 6.**
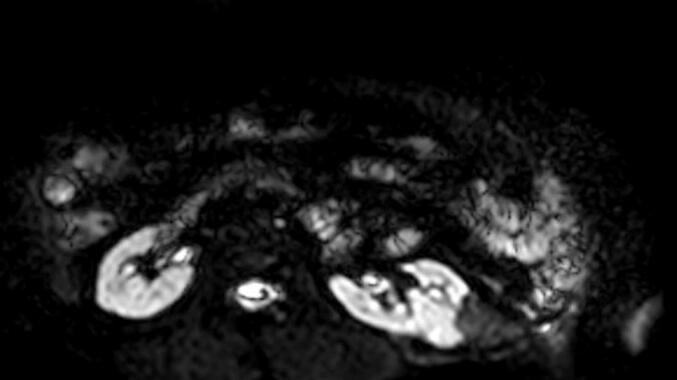

MRI findings revealed postoperative changes in the inferior pole of the kidney. In the lateral cortex of the left kidney, a 33 × 22 mm exophytic lesion was noted, which appeared isointense on T1-weighted imaging and hypointense on T2-weighted sequences, consistent with scar tissue. No restricted diffusion was observed. However, in the inferomedial surgical bed, a 5 × 9 mm T2 isointense focus with diffuse restriction and post-contrast enhancement raised suspicion for tumor recurrence. (Fig. 3., Fig. 4., Fig. 5., Fig. 6.).

Due to the incisional hernia and concern for recurrent renal cell carcinoma, surgical exploration was undertaken. The scar tissue and suspected tumor bed were completely excised. Intraoperatively, the mass was firm and adherent but lacked obvious features of malignancy.

**Fig. 7.**
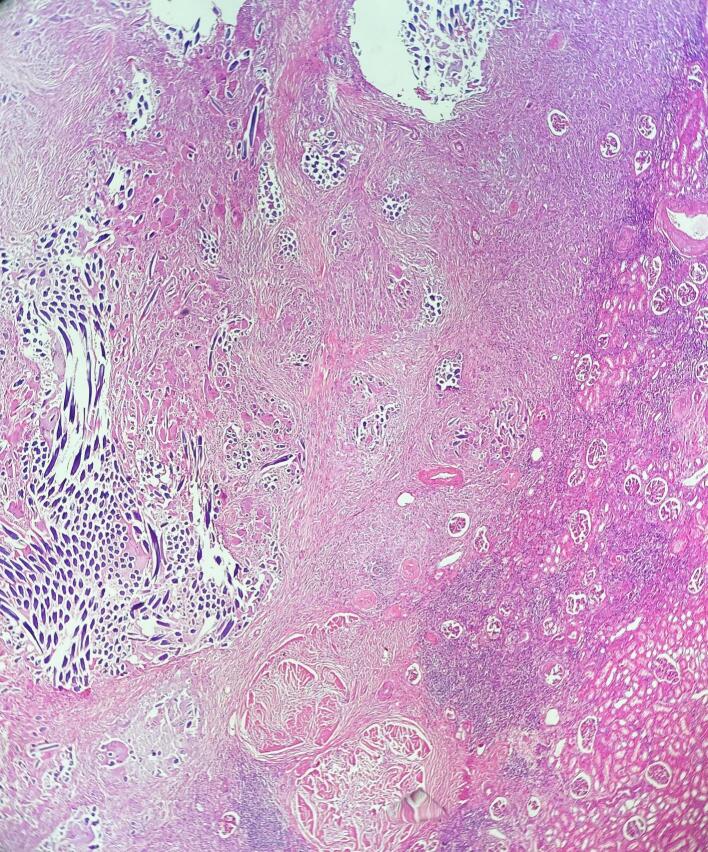

**Fig. 8.**
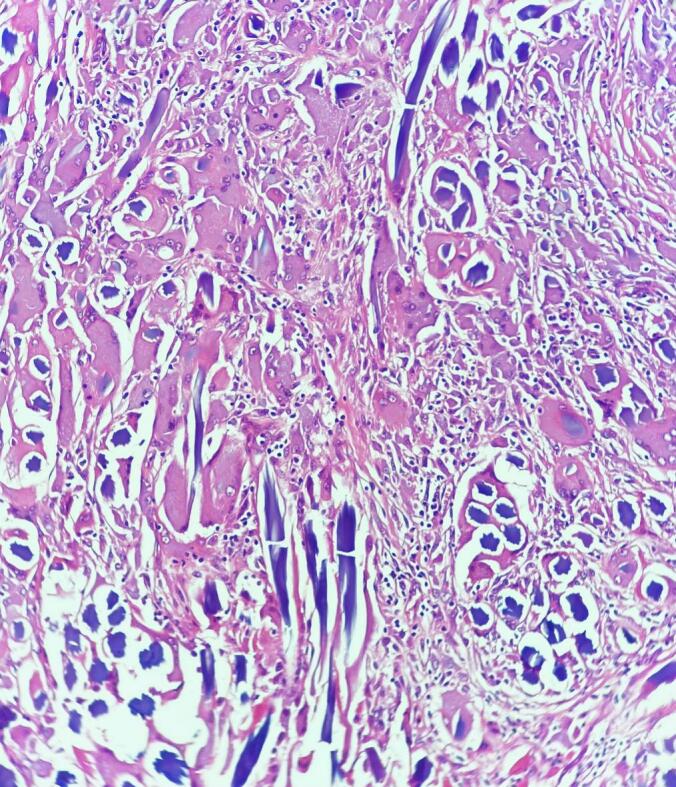

Histopathological evaluation of the resected specimen revealed a well-defined tangle of foreign material mixed with plump histiocytes and multiple foreign body type giant cells engulfing foreign bodies. Stromal fibrosis and patchy lymphocytic infiltration are noted. No malignant cells or tumor recurrences is detected. These findings were consistent with a foreign body reaction to retained oxidized cellulose (Surgicel®). (Fig. 7., Fig. 8.).

This case report has been prepared and reported in accordance with the Surgical CAse REport (SCARE) criteria to ensure completeness and transparency of clinical information [4].

## 3 Discussion

Oxidized cellulose is a commonly used hemostatic agent valued for its biocompatibility, bactericidal properties, and absorbability. Surgicel®, a commercial form, is typically resorbed within 2–6 weeks. It creates an acidic microenvironment that can intensify local inflammation and cause red blood cell lysis. This leads to the formation of acid hematin, a pigmented byproduct that may mimic tumor recurrence on imaging or histopathology, potentially complicating diagnosis [5].

Staglianò et al. reported imaging findings in three pediatric neuro-oncology patients where residual Surgicel® (oxidized regenerated cellulose) in the surgical bed mimicked tumor recurrence on postoperative MRI. In each case, areas of contrast enhancement and restricted diffusion near the resection site led to suspicion of residual or recurrent tumor. However, second-look surgeries and histopathology revealed foreign-body granulomatous reactions to Surgicel®, not neoplastic tissue. These findings highlight that Surgicel® can appear hyperintense on T2, show contrast enhancement, and even diffusion restriction, potentially misleading radiologists and clinicians. Awareness of this pitfall is crucial to avoid unnecessary interventions [6].

On postoperative CT scans, Surgicel® typically appears as areas containing trapped air bubbles. On T2-weighted MRI images, Surgicel® exhibits a short relaxation time, resulting in low signal intensity. T2-weighted MRI is the most accurate imaging modality to differentiate residual Surgicel® from hematoma, abscess, or tumor recurrence [7].

Cormio et al. reported a case involving a patient who developed a large granuloma after a combined pubovaginal sling and cystocele repair, where Surgicel® was used for hemostasis. This granuloma closely resembled ovarian cancer on imaging studies. Despite Surgicel® being designed to absorb and aid clotting, when left inside the body, it can cause foreign body reactions that mimic tumors. Imaging findings included a cystic mass with a solid component, and MRI revealed characteristic features that can help differentiate Surgicel® granuloma from malignancy [8].

Capozza et al. report a case where oxidized regenerated cellulose (Surgicel™), used as a hemostatic agent in brain surgery, caused a rare granulomatous reaction mimicking an intracranial abscess. The patient showed symptoms and imaging suggestive of infection, but surgery revealed inflammation from the material, not an abscess. This highlights the need to consider foreign body reactions in postoperative diagnoses [9].

Agarwal et al. report a Surgicel-induced granuloma mimicking local recurrence after laparoscopic nephron-sparing surgery. MRI at 3 months showed a 2.5 cm enhancing mass; biopsy confirmed foreign-body granuloma. Serial imaging over 12 months revealed lesion regression. Surgicel can cause granulomatous pseudotumors complicating postoperative assessment [10].

Hong-Wei Gao et al. report a case of a 37-year-old woman who developed an 8-cm ovarian mass one month after hysterectomy and right salpingo-oophorectomy. Histology revealed a foreign-body granulomatous reaction to oxidized cellulose (Surgicel) [11].

A thorough surgical history and multidisciplinary evaluation are essential before concluding tumor recurrence, as retained materials like Surgicel® can mimic complications on imaging. Our prior experience showed that Surgicel may appear similar to hematoma or abscess postoperatively, but can be distinguished using T2-weighted MRI. Recognizing these imaging characteristics and correlating them with clinical history helps prevent misdiagnosis and unnecessary treatments [7].

Diagnostic evaluation of solid renal masses suspected to be foreign body granulomas mimicking tumor recurrence involves a multimodal approach. Biopsy plays a critical role in obtaining histopathological confirmation, especially when radiological findings are inconclusive or atypical. Positron emission tomography/computed tomography (PET/CT) can provide additional metabolic information to differentiate benign from malignant lesions. In some cases, careful clinical follow-up with serial imaging may be appropriate to monitor lesion stability before deciding on invasive intervention [12].

To prevent misinterpretation of postoperative imaging, it is essential to maintain thorough documentation of Surgicel or other hemostatic agents used during surgery. Radiologists and urologists must be aware of the characteristic imaging appearances of these materials, as retained Surgicel can mimic hematomas, abscesses, or tumor recurrence. Enhanced interdisciplinary communication and familiarity with such postoperative findings can significantly reduce unnecessary interventions or surgeries prompted by false-positive radiologic interpretations.

## 4 Conclusion

This case underscores a critical diagnostic pitfall where retained Surgicel®, a commonly used hemostatic agent, closely mimicked local tumor recurrence following partial nephrectomy. Such presentations may lead to significant patient anxiety, unnecessary diagnostic workup, or even unwarranted surgical interventions. Accurate interpretation of postoperative imaging—supported by thorough surgical documentation and a high index of suspicion for foreign body reactions—is essential to avoid misdiagnosis. Ultimately, close interdisciplinary communication between urologists, radiologists, and pathologists is vital for optimizing postoperative care, minimizing invasive procedures, and improving patient outcomes.

## Consent

Written informed consent was obtained from the patient for publication and any accompanying images. A copy of the written consent is available for review by the Editor-in-Chief of this journal on request.

## Ethical approval

This study was reviewed by our Institutional Review Board (IRB) and was deemed exempt from formal ethical approval because the treatment and data collection were based entirely on established clinical guidelines and standard urological practice performed by an expert urologist. No experimental interventions or novel protocols were involved, and all actions followed evidence-based, routine medical care.

## Funding

There is no funding for this case report.

## Author contribution

Dr. Sina Samenezhad: study concept, data analysis, interpretation, writing the paper, data collection.

Dr. Anahita Ansari Djafari: study concept, interpretation.

Dr. Seyyed Ali Hojjati; study concept, data collection.

Dr. Azadeh Rakhshan; study concept, data collection.

Dr. Dorna Rafighi; data collection, data analysis.

## Guarantor

Dr. Sina Samenezhad.

## Conflict of interest statement

There is no conflict of interest in this case report.
